# Extrachromosomal circles of satellite repeats and 5S ribosomal DNA in human cells

**DOI:** 10.1186/1759-8753-1-11

**Published:** 2010-03-08

**Authors:** Sarit Cohen, Neta Agmon, Olga Sobol, Daniel Segal

**Affiliations:** 1 Department of Molecular Microbiology & Biotechnology Tel-Aviv University, Tel-Aviv 69978, Israel

## Abstract

**Background:**

Extrachomosomal circular DNA (eccDNA) is ubiquitous in eukaryotic organisms and was detected in every organism tested, including in humans. A two-dimensional gel electrophoresis facilitates the detection of eccDNA in preparations of genomic DNA. Using this technique we have previously demonstrated that most of eccDNA consists of exact multiples of chromosomal tandemly repeated DNA, including both coding genes and satellite DNA.

**Results:**

Here we report the occurrence of eccDNA in every tested human cell line. It has heterogeneous mass ranging from less than 2 kb to over 20 kb. We describe eccDNA homologous to human alpha satellite and the *Sst*I mega satellite. Moreover, we show, for the first time, circular multimers of the human 5S ribosomal DNA (rDNA), similar to previous findings in *Drosophila *and plants. We further demonstrate structures that correspond to intermediates of rolling circle replication, which emerge from the circular multimers of 5S rDNA and *Sst*I satellite.

**Conclusions:**

These findings, and previous reports, support the general notion that every chromosomal tandem repeat is prone to generate eccDNA in eukryoric organisms including humans. They suggest the possible involvement of eccDNA in the length variability observed in arrays of tandem repeats. The implications of eccDNA on genome biology may include mechanisms of centromere evolution, concerted evolution and homogenization of tandem repeats and genomic plasticity.

## Background

The genome of eukaryotes has been considered for a long time to be relatively stable. However, various phenomena have been described which exhibit the plasticity of the genome and occur during the normal lifespan of the organism. An intriguing manifestation of the plasticity of the eukaryotic genome is the occurrence of extrachromosomal circular DNA (eccDNA) - also termed spcDNA (small poly-dispersed circular DNA). While the population of extrachromosomal circles may include intermediates of mobile elements or viral genomes, here we refer to the circular molecules as eccDNA which are derived primarily from chromosomal repetitive sequences that do not appear to harbour an intrinsic 'jumping' or excision mechanism mediated by specific sequences.

eccDNA is ubiquitous in eukaryotic genomes and has been detected in every organism tested, including human tissues and cultured cells [1,2]. eccDNA has been reported in various human cells and was characterized mainly by electron microscopy or cloning. The latter attempted to represent the entire population of genomic circles. Using these techniques, which were laborious and provided only limited data, eccDNA was found to occur in human cells in a wide range of sizes (from several hundred base pairs to several kilobase pairs) and to consist of various chromosomal sequences including repetitive and unique sequences [2-6].

**Figure 1:**
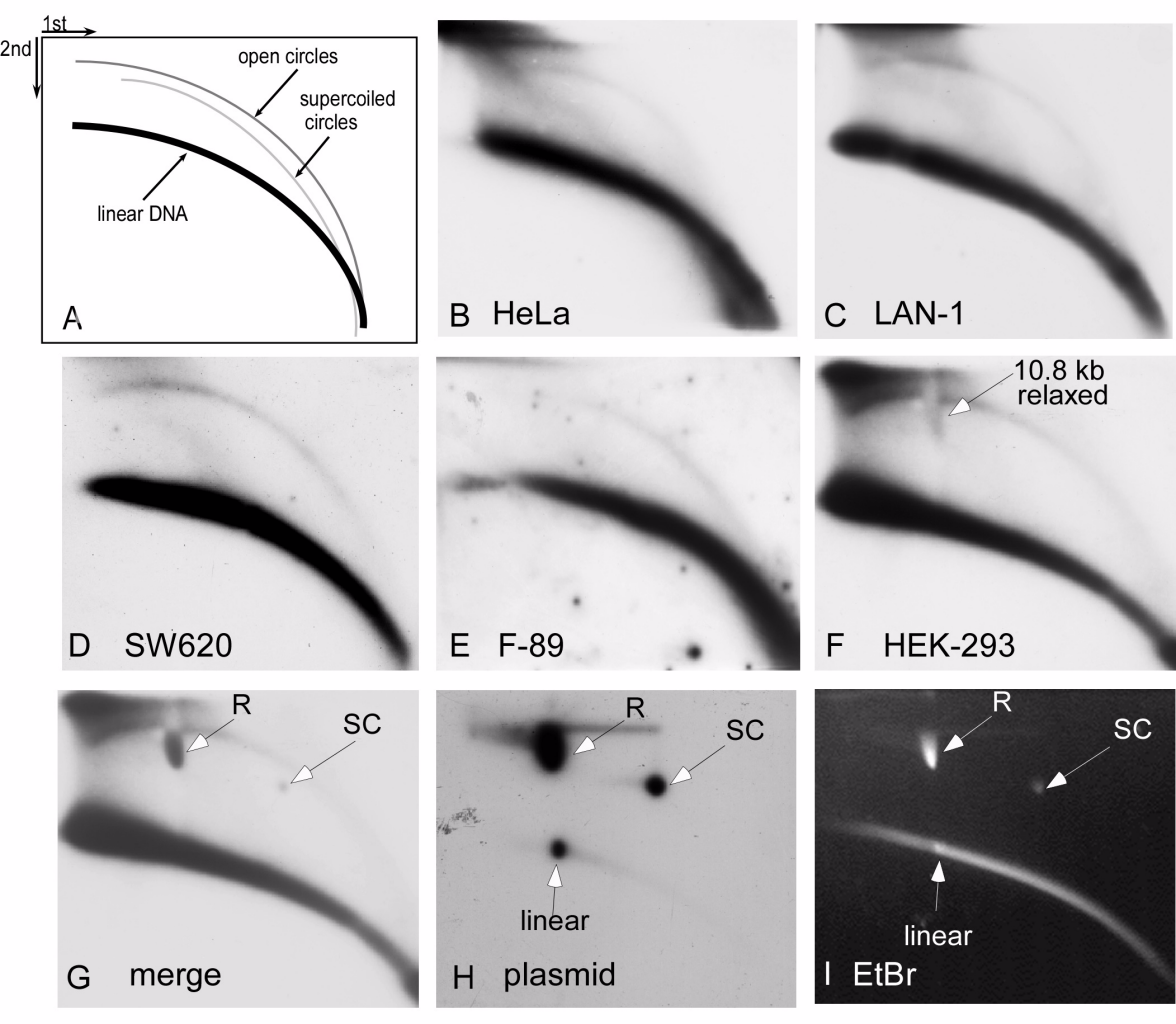
**Alpha satellite sequences in extrachromosomal circular DNA from human cell lines**. (A) A diagram of 2D gel electrophoretic patterns of genomic DNA generated by populations of linear and circular molecules heterogeneous in size. Each arc consists of molecules sharing the same structure, but differing in mass (Cohen and Lavi 1996). Hybridization with specific probes enables detection of specific sequences within the population of eccDNA. (B-E) Total DNA from human cell lines (indicated in each panel) was cleaved with *Eco*RI, digested with 'plasmid safe' DNase, analysed on two-dimensional (2-D) gels and hybridized with an 'all centromer' polymerase chain reaction product directed to the conserved region of the different types of alpha satellite. In all cases the probe hybridized with the linear DNA and with the arc that corresponds to open circles. (F-I) DNA from HEK-293 (human embryonic kidney cells) cells was mixed with a 10.8 kb plasmid prior to 2D gel analysis. Ethidium bromide (EtBr) staining of the gel reveals the three forms of the plasmid as well as the arc of linear DNA (I). These forms are visible upon hybridization with a plasmid probe (H). Hybridization with alpha satellite probe reveals the arcs of linear DNA and open circles (F). Note that the arc of open circles has a local deformation at the migration point of the over-loaded relaxed form of the plasmid. This is further confirmed by the merged image of panel F with a shorter exposure of panel H (G). White arrows indicate the plasmid forms; R = relaxed; SC = supercoiled.

**Figure 2:**
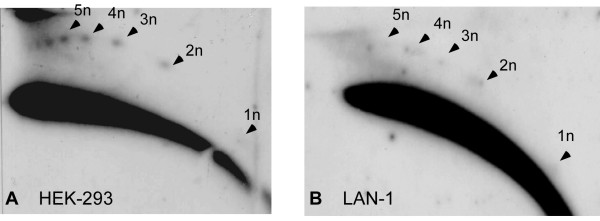
**Extrachromosomal circular multimers of the human *Sst*I satellite DNA**. Genomic DNA from HEK-293 (human embryonic kidney cells) (A) and LAN-1 (B) cells was cleaved with *Eco*RI, digested with 'plasmid safe' DNase, analysed on 2D gels and hybridized with *Sst*I probe. A ladder of discrete spots indicates open circles in the size of multiples of the 2.5 kb repeating unit. Arrowheads indicate the multimers.

To facilitate the direct detection and characterization of eccDNA within preparations of genomic DNA a two-dimensional gel (2D gel) electrophoresis was developed. This technique separates DNA molecules according to their size and structure. Thus, typical arcs are formed of molecules sharing similar structure but differing in mass (Figure 1a) [7]. Following hybridization with specific probes, arcs typical of linear DNA, supercoiled circles and open circles (it could not be determined whether these were covalently closed relaxed circles or nicked circles), can be identified. The 2D gel allows one to assess the size distribution of eccDNA, its molecular organization and its sequence content. Using this techniques eccDNA was characterized in various organisms, including rodents [7,8], *Xenopus *[9-11], *Drosophila *[12] and plants [13-15]. Chromosomal tandem repeats were found to be over-represented in the population of eccDNA. These include non-coding satellite repeats and tandemly organized coding genes, such as ribosomal DNA. Circular multimers of the repeating elements were resolved by the 2D gels in all tested organisms whenever the length of the repeat was long enough to generate a pattern of discrete spots (Figure 2; for a recent review see [1]). The formation of eccDNA was found to be independent of chromosomal DNA replication [9] but may be enhanced upon chemical stress by DNA damaging agents [7,12,16], and probably involves intrachromosomal homologous recombination and looping-out. Furthermore, rolling circle replication of eccDNA appears to occur in *Drosophila *irrespectively of the expression of the replicated genes [17].

In human cells, a 2D gel analysis of the genomic fraction enriched for low-molecular-weight DNA (Hirt extract) revealed eccDNA homologous to the highly repetitive genomic fraction Cot-1[16]. This eccDNA was found in cancerous cells (HeLa [human cervical carcinoma epithelial cells] and colon carcinoma cells) and in Fanconi anaemia cells. Normal skin fibroblasts only exhibited detectable levels of Cot-1 eccDNA after treatment with the carcinogen *N*-Methyl-*N*'-Nitro-*N*-Nitrosoguanidine (MNNG)[16]. Another important type of eccDNA found in human cells (and in other organisms) using 2D gels is the *t*-loop circles which consist of telomeric repeats [18-20]. The formation, and the possible role, of this type of circular DNA in telomere dynamics has been extensively investigated [21] and is beyond the scope of this work.

Detailed characterization of human eccDNA was halted for some years due to technical difficulties that hampered its easy detection. Two main experimental developments have prompted us to resume the characterization of human eccDNA. The first is the recent improvement of the sensitivity and resolution of 2D gels by treatment of the DNA with double stranded exonuclease, which facilitated the detection of eccDNA in DNA samples where it is barely detectable [13-15]. The second is the emerging notion that chromosomal tandem repeats are the most prominent sequences composing eccDNA in any organism tested so far [1]. Therefore, an obvious question is whether tandem chromosomal repeats are present in human eccDNA.

In this work we show that eccDNA is easily detected in both cancerous and normal human cells. These eccDNA molecules contain sequences corresponding to satellite DNA and to 5S ribosomal DNA (rDNA). In addition, intermediates of rolling circle replication are detected for 5S rDNA. These findings have wide implications for the maintenance and evolution of chromosomal tandem repeats, including processes such as repeat diminution, expansion, homogenization and mobility of tandem genes.

## Results

### Extrachromosomal circular DNA of human alpha satellite is detected by 2D gel electrophoresis

Accumulating data indicate that tandemly organized repetitive sequences are highly represented in the population of eccDNA. The first specific sequence chosen as a probe for detecting tandem repeats in eccDNA of human cells using 2D gel analysis was the highly abundant alpha satellite, which serves as a paradigm for understanding the genomic organization of tandem repeats. Alpha satellite DNA is a primate-specific family of tandemly repeated sequences present in the centromeric regions of all human chromosomes and constitutes about 3%-5% of each chromosome [22], or up to several million base pares. Its basic unit is a monomer repeat of approximately 170 bp. This monomer contains variable regions which are characteristic to the chromosome-specific variant, in addition to regions that are highly conserved among the different repeats. A polymerase chain reaction (PCR) fragment designed to detect the conserved region of the various monomer types of alpha satellite, which commonly serves as an 'all centromere' FISH (fluorescence *in-situ *hybridization) probe [23] was used to detect eccDNA in human genomic DNA (see Methods). Total genomic DNA extracted from confluent cultures of various laboratory-used human cell lines was cleaved with *Eco*RI and subjected to digestion with 'plasmid-safe' DNase to eliminate most of the linear molecules and, hence, enrich the sample for circular DNA. This treatment improves the sensitivity of the 2D gels (Additional file 1, Supplementary Fig. S1C, D) and the resolution of eccDNA analysed on such gels [13]. Electrophoresis on 2D gels and hybridization with the alpha satellite probe revealed a clear continuous arc of open circles in all tested DNA samples prepared from HeLa, LAN-1 and SW620 cells (Figure 1B-D,). While these cell lines are derived from human tumours, a question was raised about whether eccDNA can be detected also in normal or close-to-normal cell lines. DNA from HEK-293 cells and from F-89 cells was analysed as above and, in both cases, eccDNA homologous to alpha-satellite probe was detected (Figure 1E, F). Taken together, these findings suggest that alpha satellite circles occur in various human cell lines and that eccDNA might be a normal phenomenon in human cells as was found in *Xenopus *embryos, *Drosophila *and plants [11-13].

The identity of the molecules comprising the arc of eccDNA as open circles was determined by their position of migration on the 2D gel and verified by mixing a DNA sample from HEK-293 with a 10.8 kb plasmid prior to the 2D gel analysis. Although the eccDNA is not detected by Ethidium-bromide staining, the three forms of the plasmid are clearly visible (Figure 1I). It should be noted that the relatively large amount of the relaxed plasmid form caused a local deformation in the migration of the eccDNA, as revealed by the hybridization of the same blot with alpha satellite probe (Figure 1I), with the plasmid probe (Figure 1H) and the merge of the signals obtained by both probes (Figure 1G). The merge also indicates that supercoiled eccDNA was not detected on our gels, which was similar to previously reported 2D gel data of DNA from humans and other organisms [8,11-14,16]. The arc of eccDNA continues towards the high-molecular weight region of the gel, beyond the 10.8 kb plasmid marker, indicating the presence of larger circular molecules. Further assessment of the size range of the human eccDNA was obtained by mixing genomic DNA with two plasmids, 2.7 kb and 7.76 kb long (including the latter's 15.5 kb dimer) and analysing them on 2D gel. Hybridization with both an alpha satellite and a plasmid probe revealed the spots of the relaxed plasmid forms on the arc of eccDNA (Additional file 2, Supplementary Fig. S2). This arc continues towards the high-molecular-weight region of the gel, beyond the 15.5 kb marker. Longer exposures demonstrate that the arc of eccDNA can also continue towards the low-molecular-weight region below the 2.7 kb marker (not shown, but see a similar size determination of 5S rDNA circles below). These results indicate a population of eccDNA that has a heterogeneous mass, which can be smaller than 2 kb and larger than 20 kb. This size range was found for every probe tested throughout this work and is similar to the size range of eccDNA found in other organisms [11-13].

### Circular multimers of the human 2.5 kb *SstI *satellite repeat

The primate-specific *Sst*I repeat family was identified as tandemly repetitive DNA sequences consisting of 2.5 kb long repeating units [24]. Approximately 400 copies of the repeating unit are located, as tandem arrays, on chromosomes 4 (4q31) and 19 (19q13.1-q13.3). We predicted that sequences homologous to *Sst*I repeats should exist in eccDNA due to their tandem organization. This organization should make the *Sst*I repeats an appropriate substrate for eccDNA formation, if in human cells, as in many other organisms, any tandem repeat can generate circular molecules. Indeed, eccDNA homologous to the *Sst*I repeat was found when human genomic DNA was analysed on 2D gels as above (Figure 2). This eccDNA appears as a ladder pattern of distinct spots whose sizes were determined to be multiples of the 2.5 kb repeating unit using circular markers (data not shown but see a similar size determination of 5S rDNA circles below). This pattern is consistent with the organization of eccDNA as ladders consisting of multiples of tandemly repeated units, large enough to be resolved by the gel conditions, in mouse *Xenopus*, *Drosophila *and plants [8,11-14].

### *Alu *repeats were not detected in human eccDNA using 2D gels

Data from libraries of eccDNA suggested that non-tandem repeats, including unique genes and dispersed repeats such as SINES and LINES (short and long interspersed nuclear elements, respectively), may also be present in a circular form [25-28]. We predicted that, if it was indeed found in human eccDNA using 2D gel analysis, interspersed elements will probably yield eccDNA of heterogeneous size (unlike the exact multimers expected from tandem repeats) that include flanking genomic sequences, probably as a result of cis-recombination between neighbouring elements.

The most common repetitive element in the human genome is the *Alu *repeat. It is the prototype of SINES, and is the most abundant sequence in the human genome, with a copy number of about 10^6 ^per haploid genome. Its 280 bp elements are dispersed in the genome at an average distance of 3 kb.

**Figure 3:**
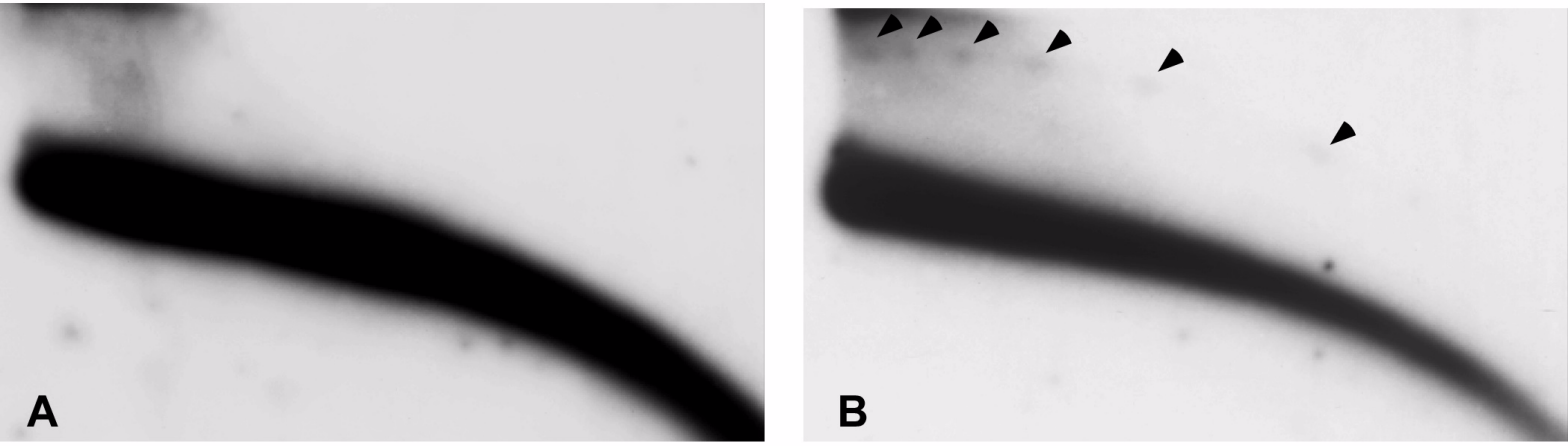
**Absence of eccDNA homologous to the dispersed *Alu *repeats**. HEK-293 (human embryonic kidney cells) DNA, was cleaved with *Eco*RI followed by 'plasmid safe' digestion and analysed on a two-dimensional gel. Hybridization of the blot with *Alu *probe revealed only the arc that corresponds to linear DNA (A). Rehybridization of the same blot with *Sst*I probe revealed both linear DNA and eccDNA multimers (B). Arrowheads indicate the circular multimers.

We did not detect *Alu *eccDNA in genomic DNA from HeLa, HEK-293 (human embryonic kidney cells) and LAN1 neuroblastoma cells (Figure 3A and data not shown) while rehybridization indicated that the same DNA samples did contain *Sst*I multimers (Figure 3B) or eccDNA homologous to alpha satellite (data not shown). Thus, the lack of eccDNA signals homologous to the *Alu *repeats suggests that this sequence is under-represented (if not absent) in a circular form compared to tandem repeats. Furthermore, this finding is consistent with previous observations in which dispersed repeats from mouse, *Xenopus *and *Drosophila *were not detected in eccDNA using 2D gels, despite their high copy number in the corresponding genomes, which is manifested by strong signals on the arc of linear DNA [8,11,12].

### 5S rDNA in human eccDNA

Ribosomal DNA (rDNA) is of special interest among the sequences present in eccDNA. It is organized in tandem arrays in a single locus or a few loci in eukaryotes. Variation in copy number is a common characteristic of rDNA [29] and has been reported in many organisms including yeast [30], *Drosophila *[31], *Arabidopsis *[32] and, recently, in humans [33]. eccDNA homologous to 5S rDNA has been detected in *Xenopus *embryos [11], *Drosophila *[12,34,35], the plant species *Arabidopsis thaliana *[13,15] and *Brachycome dichromosomatica *[13].

Human 5S rDNA consists of 2.2 kb repeating elements that include the 5S rRNA gene. These repeats are organized in tandem arrays and their copy number in normal individuals varies from 35 to 175 copies per haploid genome - although the human genome project estimated only 17 repeats, probably due to the known difficulties in the sequencing and assembly of tandem arrays [33]. In their study, Stults *et al*. provided evidence for meiotic and somatic recombination within the 5S rDNA arrays that could be inter- or intra-chromosomal [33]. Hence, we found it intriguing to test whether eccDNA homologous to 5S rDNA does exist in humans.

**Figure 4:**
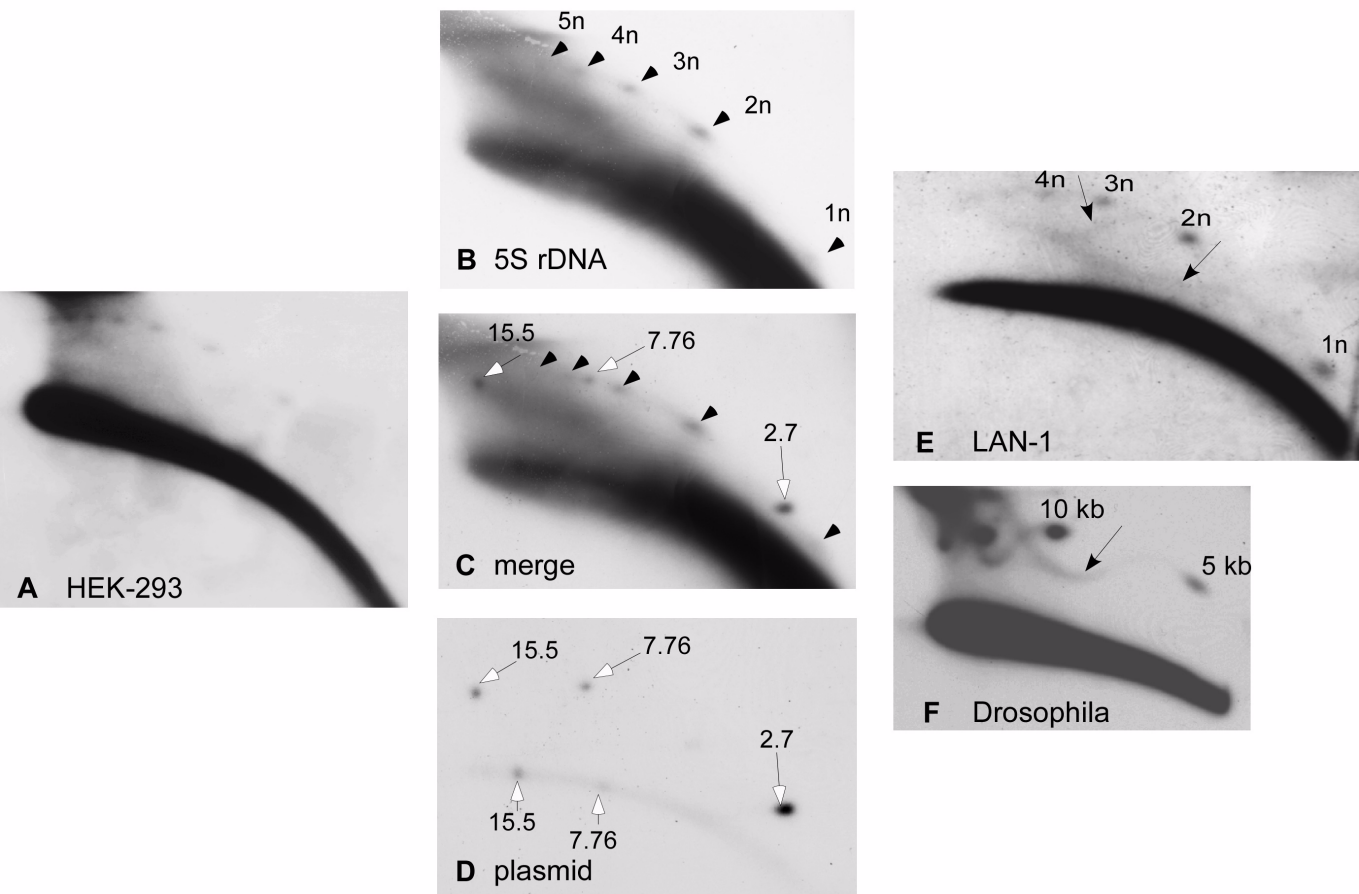
**Circular multimers of 5S rDNA and their rolling circle intermediates**. Genomic DNA from HEK-293 (human embryonic kidney cells) digested with *Eco*RI and 'plasmid safe' DNase, was analysed on two-dimensional (2D)gel and hybridized with 5S rDNA probe. A series of spots forming a ladder indicate the presence of extrachromosomal circular multimers of the repeat (A). (B-D) For size analysis of the multimers, HEK-293 DNA digested with *Hind*III and 'plasmid safe', was mixed with plasmids of 2.7 kb, 7.76 kb and a 15.5 dimer of the latter prior to 2D-gel analysis. The blot was hybridized with 5S rDNA, revealing the circular multimers (B) and then rehybridized with a plasmid probe revealing the linear and the relaxed forms of the plasmids (D). (C) Merging the two autoradiograms verified that the eccDNA was indeed in the size of 5S rDNA multiples (see text). Arrowheads indicate multiples of 5S rDNA, and white arrows indicate plasmid markers. (E-F) Intermediates of rolling circle replication emerge from eccDNA. (E) Genomic DNA from LAN-1 cells, digested with *Eco*RI and 'plasmid safe', was analysed on 2D gel and hybridized with 5S rDNA probe. Long exposure reveals sigmoid arcs emerging from the 1n and 2n eccDNA (black arrows). Similar sigmoid arcs were found emerging from circular multimers of the 5 kb histone gene cluster of *Drosophila *(F), and were identified as rolling circle intermediates [17].

A 5S rDNA specific probe, which recognizes exclusively the 5S rDNA array (and neither the repetitive elements that are included within the 2.2 kb element nor the dispersed pseudogenes), has been previously designed [33]. Using this probe we detected eccDNA organized as a ladder of up to eight circular multimers of 5S rDNA in DNA prepared from HEK-293, LAN-1 and HeLa cells (Figure 4 and data not shown).

In order to verify the sizes of the multimers, we used the plasmid markers described above which co-migrated with the genomic DNA on the 2D gel. Hybridization with 5S rDNA or plasmid probes revealed the spots of the relaxed plasmid forms on the arc of eccDNA (Figure 4C). The presence of two 5S rDNA spots between the 2.7 and the 7.76 kb markers is consistent with the expected sizes of dimer (4.4 kb) and trimer (6.6 kb) forms of the 2.2 kb repeat. In addition, the smallest faint spot homologous to 5S rDNA is smaller than the 2.7 kb marker as expected from a monomer size circle.

### Putative rolling circle intermediates of human 5S rDNA

A long exposure of membranes probed with 5S rDNA revealed specific continuous sigmoid arcs, emerging from the circular multimers and extending toward higher molecular weights in LAN-1 cells (Figure 4E). Similar patterns were also observed in HEK-293 DNA following long exposures of *Sst*I probed membranes (Additional file 3, Supplementary Fig. S3). These sigmoid arcs have been previously detected in circular multimers of several tandem repeats in *Drosophila *including the 5 kb histone cluster (Figure 4F). Based on their migration, which is comparable to different known cases of rolling circle replication, and their co-purification with chromosomal replication intermediates (on benzoylated naphthoylated diethylaminoethyl (BND)-cellulose), the molecules that comprise the sigmoid arcs were identified as rolling circle intermediates (RCIs) [17].

The observed signals of the putative RCIs were much weaker than those obtained in *Drosophila *where eccDNA, in general, is much more abundant and can be readily detected in the absence of 'plasmid safe' treatment. However, our findings suggest that rolling circle replication of eccDNA homologous to 5S rDNA and to *Sst*I satellite might also occur in human cells.

The putative RCIs were visible in human DNA following treatment with 'plasmid safe' exonuclease although one would expect the tails of these RCIs to be sensitive to this enzyme. As mentioned above, this treatment is necessary in order to visualize eccDNA in human DNA preparations where, without the exonuclease treatment, eccDNA could barely be detected. In our study (and tat of other laboratories [14,15]) this enzyme never worked to completion, as indicated by the linear DNA left after digestion. In addition, some specific molecules of linear DNA are resistant to the enzyme, including the linear form of a marker plasmid (Additional file 1, Supplementary Figure S1).

Furthermore, in *Drosophila*, the signal of histone cluster RCIs, which is detectable also without the 'plasmid safe' exonuclease treatment, did not lose its intensity after exonuclease treatment. On the contrary, the signal became even stronger upon exonuclease treatment (Additional file 1, Supplementary Figure S1). It is possible that RCIs are trapped in the bulk of chromosomal DNA and, hence, are not completely resolved on the 2D gels. Following the elimination of the most of chromosomal DNA, RCIs are released and their signals are increased.

We have previously shown that these tails of RCIs are not single stranded but are double stranded [17]. Thus, we are convinced that, in spite of their peculiar resistance to 'plasmid safe' exonuclease, these are double stranded RCIs.

## Discussion

In this work we utilize 2D gel electrophoresis to identify eccDNA in various human cells using specific chromosomal repetitive sequences as probes. We demonstrate for the first time the presence of eccDNA homologous in the tandemly arranged 5S rDNA and the *Sst*I mega satellite, as well as eccDNA homologous to a general alpha satellite probe. eccDNA molecules are heterogeneous in mass, having a size range similar to that found for eccDNA in animal and plant systems. The sizes of eccDNA detected in this report are, in fact, dictated by the resolution limits of the 2D gels and it is conceivable that larger and smaller circular molecules also exist. Hence, we estimate the size of human eccDNA to range from less than 2 kb to over 20 kb.

This research did not aim to generate quantitative data on eccDNA in human cells, as assessments on the amount of eccDNA, based on studies with electron microscope, have been previously summarized [2]. These studies estimate that human cells contain from tens to several hundred circular molecules per cell, depending on the cell type and its physiological conditions (for example, cell cycle phase, growth conditions and stress). In the future it should be possible to identify changes in the level of eccDNA under different experimental conditions using 2D gels, after standardizing the DNA samples as previously demonstrated for plant DNA [15].

### Circular multimers of tandemly repeated elements

The occurrence of a spot-like pattern of the 5S rDNA and *Sst*I satellite (Figures 2 and 4), indicates that in humans, as in other organisms, eccDNA is composed of circular multimers of the tandemly repeated units. The same multimer organization of eccDNA was found in all organisms tested [1], which suggests a common mechanism of eccDNA formation that would involve intra-chromosomal homologous recombination and the looping-out of circular molecules (for detailed discussions, see [1,12]). Thus, the machinery of eccDNA formation appears to be universal among eukaryotes.

In this study, we have not attempted to identify eccDNA homologous to the human 45S rDNA, since its monomer size (43 kb) is too large to be detected by 2D gels. However, it is conceivable that circles composed of monomers larger than 20 kb may also exist. In *Drosophila*, eccDNA homologous to the 18/28S rDNA, whose monomer size is variable (due to a variable intergenic spacer) but is at least l0 kb long, was identified as a heterogeneous short arc at the high-molecular-weight region of the 2D gel [12]. This suggests that, similar to the 5S rDNA, the other tandemly arranged rDNAs can also appear in eccDNA.

Recently, a genome-wide survey of the largest tandemly repeated DNA families in the human genome has been published [36] and it may serve as a valuable resource of sequences that can be tested for their occurrence in eccDNA.

### Alpha satellite in eccDNA

eccDNA homologous to an all centromere probe directed to all variants of alpha satellite is readily detected in every human cell line tested. The 2D gel conditions used in this study can resolve circular multiples of elements larger than 300 bp. Hence the expected multimers of 170 bp of this repeat would migrate as a continuous arc. However, since every tandem repeat tested so far was found in eccDNA and, whenever the length of its monomer was long enough, its corresponding circular multimers were identified [1], we predict that eccDNA of variants of alpha satellite might also be organized in this manner. Indeed, higher-order-repeat (HOR) elements, composed of one to 12 alpha-satellite related (alphoid) basic monomers were found as circular multimers in a supercoiled DNA preparation from HeLa cells [37,38]. For example, circular multimers of an 849 bp element of the *Sau*3A alphoid family, which is composed of five 171 bp alphoid elements that share an average homology of 73%, were reported. These are assumed to result from a recombination between homologous monomers from each 849 bp HOR. In addition, less abundant circular forms, the size of multiples of 170 bp, were also detected which represents a recombination between any of the alphoid monomers [37,38]. Due to the high frequency of circular multimers of this 849 bp element, it was presumed to be extremely prone to excision from the chromosome. It would be interesting in the future, to expand the characterization of alpha satellite eccDNA. For example, we could probe eccDNA with specific variants of alpha satellite and characterize multiples of specific HORs. Such an approach may have wide implications on elucidating the mobility and evolution of alpha satellite and may shed light on understanding the formation of new chromosomal loci of alpha satellite including neocentromeres.

### Circles of *Alu *repeat are under-detected by 2D gels

The tandem organization of the DNA elements appears to be a pre-requisite for forming eccDNA. Dispersed elements have not been detected by 2D gels within eccDNA from the different organisms tested so far [8,12,39]. Furthermore, *Xenopus *activated egg extract could form eccDNA *de novo*, from a recombinant substrate organized in tandem repeats but not from the non tandemly arranged bacteriophage lambda DNA [9]. The under-detection of *Alu *repeats within eccDNA indicates that, if circular molecules that include *Alu *repeats do exist, their proportion in the total chromosomal copies of this sequence is relatively low. Although dispersed elements, including *Alu*, were reported in libraries of eccDNA [2], the common cloning techniques used for their construction were sensitive to various experimental conditions, which made them less representative with respect to the sequence content and organization of eccDNA (for detailed discussion see [1]). Hence, in spite of its limited sensitivity, the 2D gel electrophoresis is currently the only direct way to determine whether a sequence of interest is present in a circular form.

Considering the relatively short average distance between genomic *Alu *elements (about 3 kb), one would expect recombination between adjacent elements to generate circles, provided these elements are present in a direct repeat orientation. The under-detection (or even absence) of *Alu *repeats from the population of eccDNA, in spite of their high occurrence in the genome, may be explained if they act as 'cold spots' for meiotic and mitotic recombination, as was shown for yeast Ty elements which are the main family of dispersed repeats in *S. cerevisiae *[40,41]. The compact chromatin structure of the Ty element was shown to prevent the initiation of recombination, hindering the potentially lethal consequences of exchanges between repeated sequences at nonallelic locations [42]. Similarly, it is likely that the *Alu *repeats are also protected from inter-repeats recombination and, thus, are not found in the eccDNA population. Supporting this possibility, hyper methylation of Histone H3 lysine 9, which is usually correlated with a closed chromatin structure, has been demonstrated at *Alu *element [43].

Interestingly, a novel human mega-satellite containing amplified patterns of interspersed transposable elements has been recently described [36]. If eccDNA is derived from such tandemly arranged mega-satellites, then DNA circles should include the amplified transposable elements. By this means, DNA elements, which are normally dispersed in the genome, may appear in eccDNA.

### Rolling circle replication of human eccDNA?

In this work we demonstrate, for the first time, evidence of the likely occurrence of intermediates of RCIs of eccDNA in human cells. These are manifested on the 2D gels as specific sigmoid patterns emerging from the spots of circular multimers of 5S rDNA and *Sst*I satellite (Figures 4 and Additional file 3, Supplementary Figure S3). We have previously identified similar RCIs emerging from the circular multimers of three tandemly arranged *Drosophila *coding genes [17]. These were more intense than the putative human RCIs, which is in agreement with the high abundance of eccDNA in *Drosophila*. eccDNA, in general, and RCIs, in particular, could be easily detected in various preparations of genomic DNA from *Drosophila *tissues and cultured cells [17]. Accordingly, *Drosophila *RCIs could be enriched on a specific BND-cellulose column along with chromosomal replication forks, supporting their identification as replication intermediates. However, this enrichment was laborious and, due to the relative scarcity of eccDNA in the human cells tested in the present work, it was not feasible to apply such enrichment to the tested DNA preparations. It will be important, in the future, to search for cell types and define growth conditions that exhibit increased levels of RCIs in order to further characterize them. The mechanism of rolling circle replication (RCR) has not yet been studied. However, some hints towards the possible machinery for DNA synthesis on eccDNA, including the participation of DNA polymerases α and δ, have previously been detailed [1,9]. It might be advantageous that further mechanistic and molecular characterizations of RCR be first carried out in *Drosophila*, where the phenomenon appears to be enhanced, and then translated to human cells.

eccDNA molecules harbouring a sigmoid form (circles with hanging tails) from mung-bean were observed by electron microscopy [44], suggesting that RCR may also occur in plants. These findings, combined with putative RCIs in *Drosophila *and human cells, may imply that RCR occurs universally in eukaryotes.

RCIs are detected throughout the life cycle of *Drosophila *[17], hence they represent a normal phenomenon. Furthermore, RCR of eccDNA occurs irrespectively of the expression state of the amplified genes. This is unlike the reported cases of developmentally regulated gene amplification, such as the amplification of ribosomal genes in amphibian oocytes and of *Drosophila *chorion genes. RCR of eccDNA is also distinct from the amplification of genes that promote cancer. In both developmentally-related and cancer-associated examples, the amplification occurs only in specific cells and the amplified genes are then over-expressed. Instead, RCR of eccDNA may contribute to the accumulation of extra-copies of certain sequences that are included in the population of eccDNA. This would imply that eccDNA sequences may escape the constraint of replicating once, and only once, during a cell cycle, which is true for the tightly regulated chromosomal replication. Still, it is not clear what is the fate of the RCR products or whether the resultant newly formed concatamers can reintegrate into the chromosome. However, the formation of a concatamer with identical repeats by RCR invokes the attractive conjecture of its possible role in homogenization of tandem repeats. We speculate that integration of extrachromosomal concatamers into the chromosomes may occur alongside, or in combination with, unequal crossing-over and gene conversion, which are currently the accepted mechanisms to explain the concerted evolution of repetitive DNA [29,45].

### eccDNA and size variability of arrays of tandem repeats

The occurrence of eccDNA is intriguing with respect to the copy number variability observed in many types of tandem repeats, both satellite and rDNA arrays. For a long time expansion, diminution and homogenization of tandem repeats were explained by inter-chromosomal recombination events such as unequal crossing-over and gene conversion (for recent reviews see [29,46]). However, mathematical calculations have demonstrated that these mechanisms were not sufficient to account for the high rate of variation observed in tandem arrays [47]. Hence, along with the inter-chromosomal recombination, the involvement of circular DNA has been hypothesized: excision of eccDNA may shorten arrays of tandem repeats while their possible integration into homologous sites in the chromosome could expand the cluster. In this work, and in previous reports, we have demonstrated evidence supporting this hypothesis. *In vitro *experiments have shown that eccDNA is formed *de novo *in *Xenopus *egg extracts, even in the absence of any DNA synthesis [9,11]. This indicates that circles excised from the chromosomal substrate and, hence, generated a deletion of several repeats. We propose that excision of eccDNA may serve as a balancing mechanism protecting against the over-expansion of tandem arrays and for the maintenance of genome size. On the other hand, the homologous integration of eccDNA, or of RCR products, may expand chromosomal arrays. For example, since eccDNA is detected in somatic cells it can explain the somatic variation observed in the size of ribosomal arrays [29,33,48]. Furthermore, given that circular plasmid constructs commonly used for transformation readily integrate into the eukaryotic nuclear genome via illegitimate recombination, it is likely that a similar process could operate to reintegrate eccDNA. Therefore, eccDNA could serve as a DNA reservoir contributing to the genetic variability of genomes. In addition, integration of eccDNA may form new chromosomal loci of tandem repeats. These might be later detected as 'orphons' (genes located outside the main chromosomal locus) derived from tandem repeats and found in various organisms [49-52].

## Conclusions

Our identification of eccDNA in human cells has opened up a new research area that could potentially uncover mechanisms underlying complex behaviours of large genomes. Further research will provide an insight into the contribution of eccDNA to the genome function and dynamics, in particular with respect to genome instability, function of satellite sequences and evolution of chromosomes.

## Methods

### Human cell lines

HeLa, HEK-293 and F-89 human primary human diploid fibroblasts [53] (courtesy of Y Shiloh) were grown in Dulbecco's Modified Eagle Medium (DMEM), supplemented with 10% FCS (20% for F-89) 100 units/mL penicillin, 0.1 mg/mL streptomycin at 37°C in the presence of 5% CO_2_.

SW620 - metastatic colon carcinoma cells [54](courtesy of I Witz) - were maintained in Leibovitz L-15 medium supplemented with 15% FCS, 2 mmol/L L-glutamine, 100 units/mL penicillin, 0.1 mg/mL streptomycin, 12.5 units/mL nystatin, 10 mmol/L HEPES buffer, and 0.075% sodium bicarbonate [55].

LAN-1-human neuroblatoma cells were grown in RPMI 1640 with 10% FCS, at 37°C in the presence of 5% CO_2 _(courtesy of Y Kloog).

### *Drosophila *cells

Schneider 2 R+ (SR+) cells were grown in Schneider medium with L-Glutamine (Biological Industries) supplemented with 10% heat inactivated fetal bovine serum (Gibco), 100 unit/ml penicillin and 0.1 mg/ml streptomycin (Biological Industries) in 25 cm^2 ^flasks at 25°C, without CO_2 _regulation.

### DNA preparation

Total genomic DNA from confluent cultures was prepared as previously described [17]. The DNA (several to a few tens of micrograms) was cleaved with a restriction enzyme that did not cleave within the sequences of interest (*Eco*RI or *Hind*III, as indicated in the legends to the figures). Restriction digested DNA samples were extracted with phenol: chloroform and precipitated with ammonium-acetate and ethanol. DNA was resuspended in water and digested with 'plasmid-safe' adenosine triphosphate (ATP) dependent DNase (Epicentre) according to the manufacture's protocol. 'Plasmid-safe' DNase was used to enrich DNA samples for circular molecules using its specific affinity for double stranded linear DNA. The DNA was then either directly loaded onto the first dimension of the 2D gel or concentrated by ethanol precipitation and then resuspended and loaded onto the gel.

### Neutral-neutral 2D gel electrophoresis, blotting and hybridization

Neutral-neutral 2D gel electrophoresis, blotting and hybridization were performed as previously described [12]. Briefly, the DNA was separated on the first dimension in 0.4% agarose at 0.7 V/cm in 1× TBE buffer (Tris -Borate-EDTA) overnight, and the second dimension was in 1% agarose containing 0.3 μg/ml EtBr at 4 V/cm in 1× TBE for 4 hours. The gels were blotted onto positively charged nylon membrane (Zeta-probe, BioRad). Probes were labelled by a random priming kit (Biological Industries). Radiolabelled DNA was detected by autoradiography.

### DNA probe preparation

All probes were generated by polymerase-chain reaction (PCR) carried out on genomic DNA from HeLa cells and purified by a High Pure PCR product purification kit (Roche).

Alpha satellite probe was generated using primers directed to the conserved region of the alphoid monomer in PCR conditions as described [23]. For 5S rDNA probe we used primers as previously described [33]. *Sst*I probe (232 bp) was generated using primers designed according to its reported sequence [GneBank: X04912]: 5'GTGGTGGTGCATGGCCCCC3' and 5'GAGCTCCAGGATCACCACAGC3'.

*Alu *probe (158 bp) was generated using primers designed according to its reported consensus sequence [GneBank: U14568]: 5'GGCGGGCGGATCACGAGGTCAG3' and 5'CCCGGGTTCATGCCATTCTCCTG3'.

## Abbreviations

ATP: adenosine triphosphate; BND benzoylated naphthoylated DEAE; HEK: human embryonic kidney cells; HeLa: human cervical carcinima epithelial cells; HOR: higher order elements; PCR: polymerase chain reaction; RCI: rolling circle intermediates; RCR: rolling circle replication; SINES and LINES: short and long interspersed nuclear elements; TBE: Tris-Borate-EDTA; 2D gel: two-dimensional gel

## Competing interests

The authors declare that they have no competing interests.

## Authors' contributions

SC designed, coordinated and performed all of the experiments in the laboratory of DS. NA prepared genomic DNA, designed the specific probes and performed the initial experiments of the project. OS initiated the use of exonuclease to improve DNA analysis and participated in DNA electrophoresis and hybridizations. DS participated in the design and coordination of the project. SC and DS wrote the manuscript and NA critically commented it. All authors read and approve the final manuscript.

## Supplementary Material

Additional file 1 **Supplementary Figure S1**. Resistance of Rolling circle intermediates (RCIs) to "Plasmid Safe" nuclease. *Drosophila *genomic DNA was cleaved with *Pvu*II, mixed with the three forms of a 10.8 kb marker plasmid (A, -DNase), and half of the sample was treated with "Plasmid Safe" nuclease (B, +DNase). While most of the genomic DNA was digested, the circular plasmid forms (supercoiled (sc) and relaxed) were resistant as expected, yet the linear plasmid form was resistant to the "Plasmid Safe" as well. Blotting and hybridization with histone H3 probe revealed at short exposure (3 hours), clear multimers of 5 KB eccDNA of the histone cluster (arrowheads) in the sample treated with "Plasmid Safe" (D), while these were barely detected in the untreated sample (C). This apparent increase in the amount of detectable eccDNA following nuclease treatment is accounted for by the release of circular molecules that were trapped within the bulk of chromosomal DNA. At long exposure (3 days), RCIs (black arrows) were detected in both samples, but were more intense in the "Plasmid Safe" treated sample (F) compared to the untreated sample (E). This finding demonstrates that treatment with "Plasmid Safe" nuclease did not destroy RCIs, rather it increased their signal by facilitating their separation from linear molecules and migration on the 2D gel. Click here for file

Additional file 2 **Supplementary Figure S2**. Size analysis of alpha satellite eccDNA. Genomic DNA from HEK-293 cells was mixed with circular plasmid markers of 2.7 kb, 7.76 kb and the 15.5 kb dimer of the latter prior to 2D-gel analysis. The blot was hybridized with alpha satellite probe (A), and subsequently rehybridized with a plasmid probe (data not shown). (B) Merging the two autoradiograms indicates that the eccDNA co-migrates with the relaxed plasmid forms and its size can be larger than 15.5 kb and smaller than 2.7 kb. Black arrows indicate relaxed plasmid forms. White arrow indicates the supercoiled (sc) form of the 7.76 kb plasmid. Click here for file

Additional file 3 **Supplementary Figure S3**. Rolling circle intermediates of *Sst*I eccDNA. Longer exposure of the *Sst*I probed blot, shown in Figure 3B, revealed rolling circle intermediates emerging from the circular multimers (A). Rolling circle intermediates of the multimers of the Drosophila 5 kb histone cluster are shown as reference (B). Click here for file
