# Supplementary figures and images for: Extrachromosomal circles of satellite repeats and 5S ribosomal DNA in human cells
Source: Mob DNA. 2010 Mar 8;1:11. doi: 10.1186/1759-8753-1-11 (PMC3225859; doi:10.1186/1759-8753-1-11)

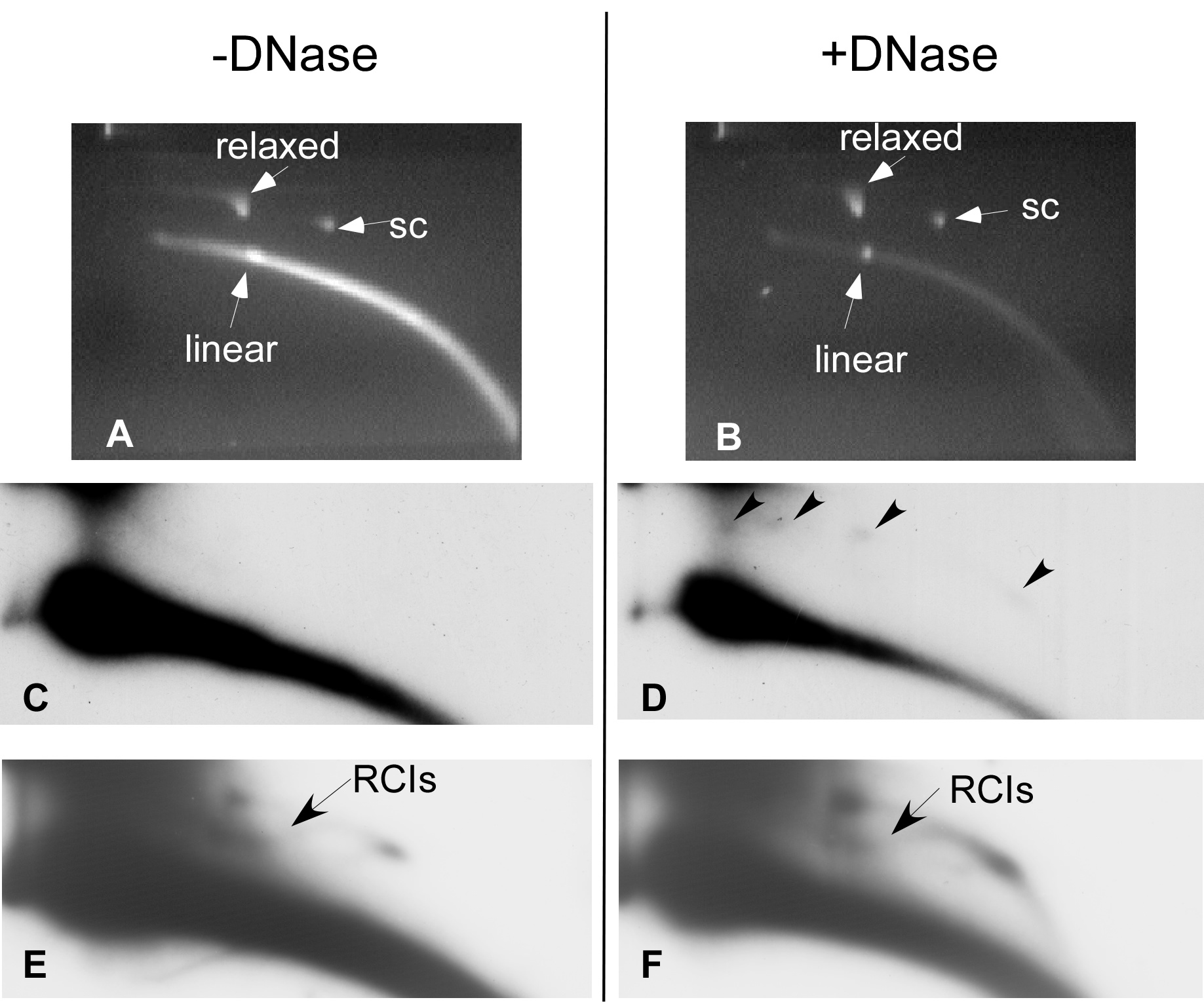

Supplement: Additional file 1 — Supplementary Figure S1. Resistance of Rolling circle intermediates (RCIs) to "Plasmid Safe" nuclease. Drosophila genomic DNA was cleaved with PvuII, mixed with the three forms of a 10.8 kb marker plasmid (A, -DNase), and half of the sample was treated with "Plasmid Safe" nuclease (B, +DNase). While most of the genomic DNA was digested, the circular plasmid forms (supercoiled (sc) and relaxed) were resistant as expected, yet the linear plasmid form was resistant to the "Plasmid Safe" as well. Blotting and hybridization with histone H3 probe revealed at short exposure (3 hours), clear multimers of 5 KB eccDNA of the histone cluster (arrowheads) in the sample treated with "Plasmid Safe" (D), while these were barely detected in the untreated sample (C). This apparent increase in the amount of detectable eccDNA following nuclease treatment is accounted for by the release of circular molecules that were trapped within the bulk of chromosomal DNA. At long exposure (3 days), RCIs (black arrows) were detected in both samples, but were more intense in the "Plasmid Safe" treated sample (F) compared to the untreated sample (E). This finding demonstrates that treatment with "Plasmid Safe" nuclease did not destroy RCIs, rather it increased their signal by facilitating their separation from linear molecules and migration on the 2D gel. [file 1759-8753-1-11-S1.JPEG]

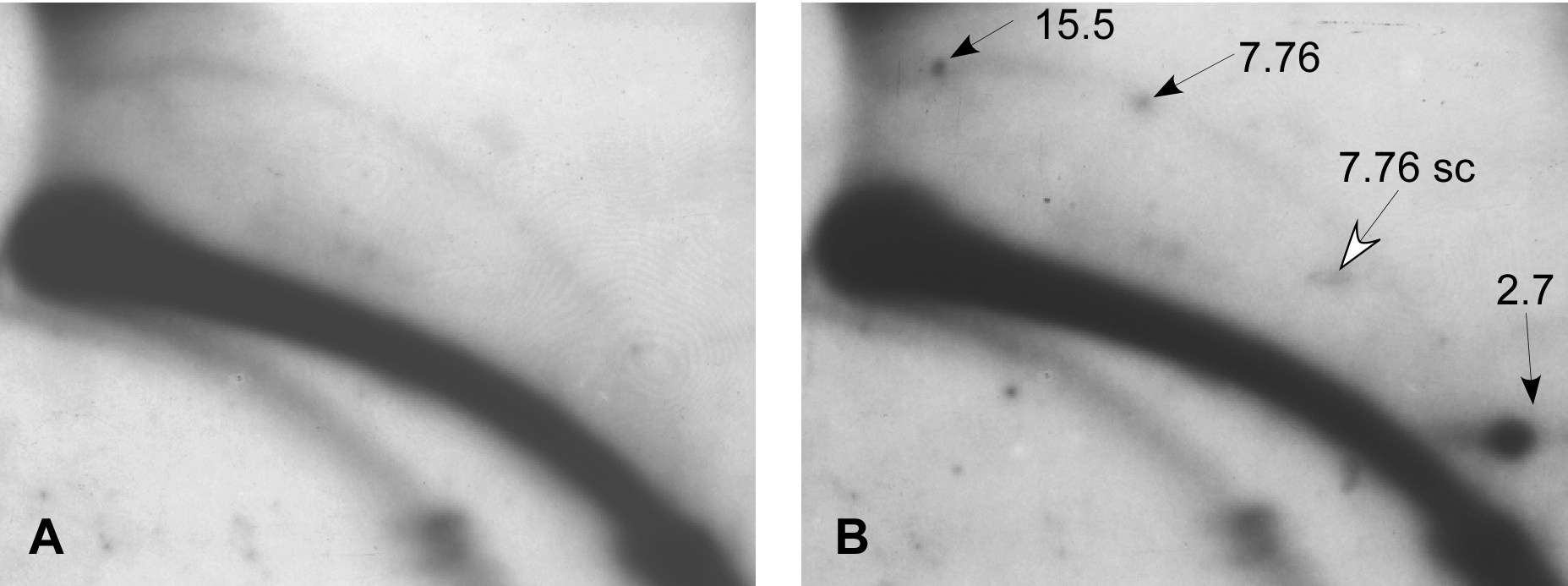

Supplement: Additional file 2 — Supplementary Figure S2. Size analysis of alpha satellite eccDNA. Genomic DNA from HEK-293 cells was mixed with circular plasmid markers of 2.7 kb, 7.76 kb and the 15.5 kb dimer of the latter prior to 2D-gel analysis. The blot was hybridized with alpha satellite probe (A), and subsequently rehybridized with a plasmid probe (data not shown). (B) Merging the two autoradiograms indicates that the eccDNA co-migrates with the relaxed plasmid forms and its size can be larger than 15.5 kb and smaller than 2.7 kb. Black arrows indicate relaxed plasmid forms. White arrow indicates the supercoiled (sc) form of the 7.76 kb plasmid. [file 1759-8753-1-11-S2.JPEG]

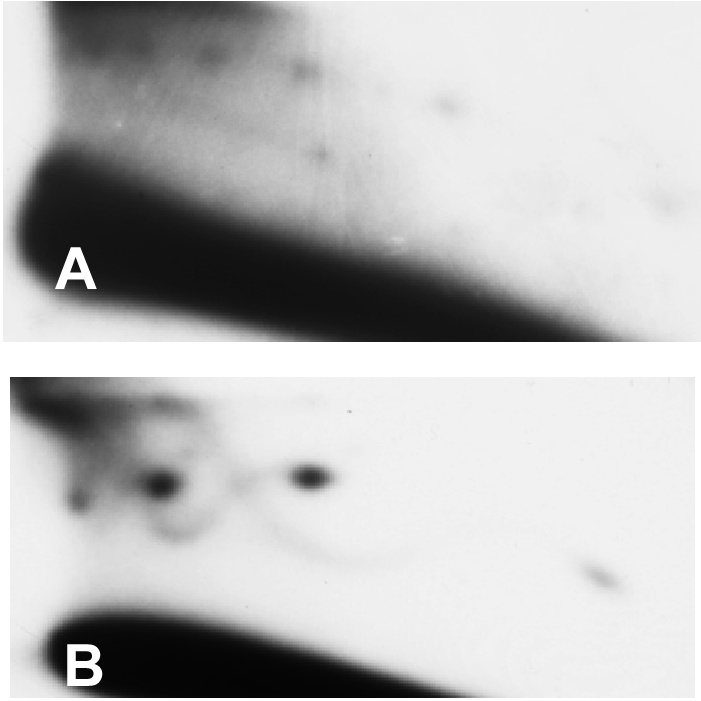

Supplement: Additional file 3 — Supplementary Figure S3. Rolling circle intermediates of SstI eccDNA. Longer exposure of the SstI probed blot, shown in Figure 3B, revealed rolling circle intermediates emerging from the circular multimers (A). Rolling circle intermediates of the multimers of the Drosophila 5 kb histone cluster are shown as reference (B). [file 1759-8753-1-11-S3.JPEG]
